# Supplementary material for: Adipose Tissue-Derived Extracellular Vesicles Contribute to Phenotypic Plasticity of Prostate Cancer Cells
Source: Int J Mol Sci. 2023 Jan 8;24(2):1229. doi: 10.3390/ijms24021229 (PMC9864182; doi:10.3390/ijms24021229)
Supplement: Supplementary file 1 [file ijms-24-01229-s001.zip › Supplemental Figures.pdf]

Supplemental Figure S1

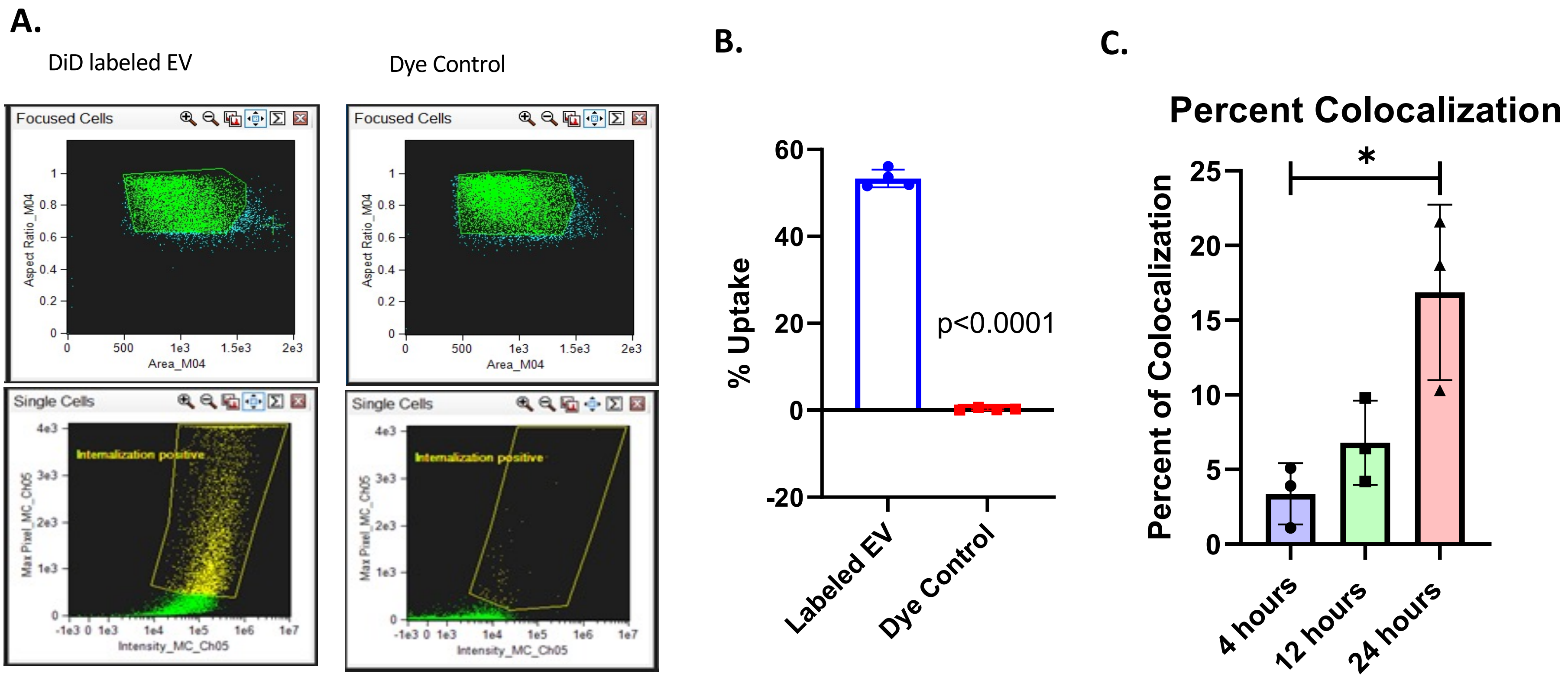

Supplemental Figure S1: EV labeling and colocalization. A and B: EV uptake and cell cycle phase were assessed using Amnis ImageStream. Double ultracentrifugation eliminated nearly all DiD aggregate background fluorescence as shown by flow cytometry gating and quantification of uptake in PC3ML incubated with labeled EVs and a dye (DiD) only negative control. **C**: Colocalization analysis revealed that after 12 hours incubation, less than 10% EV appear in the lysosome.

Supplemental Figure S2

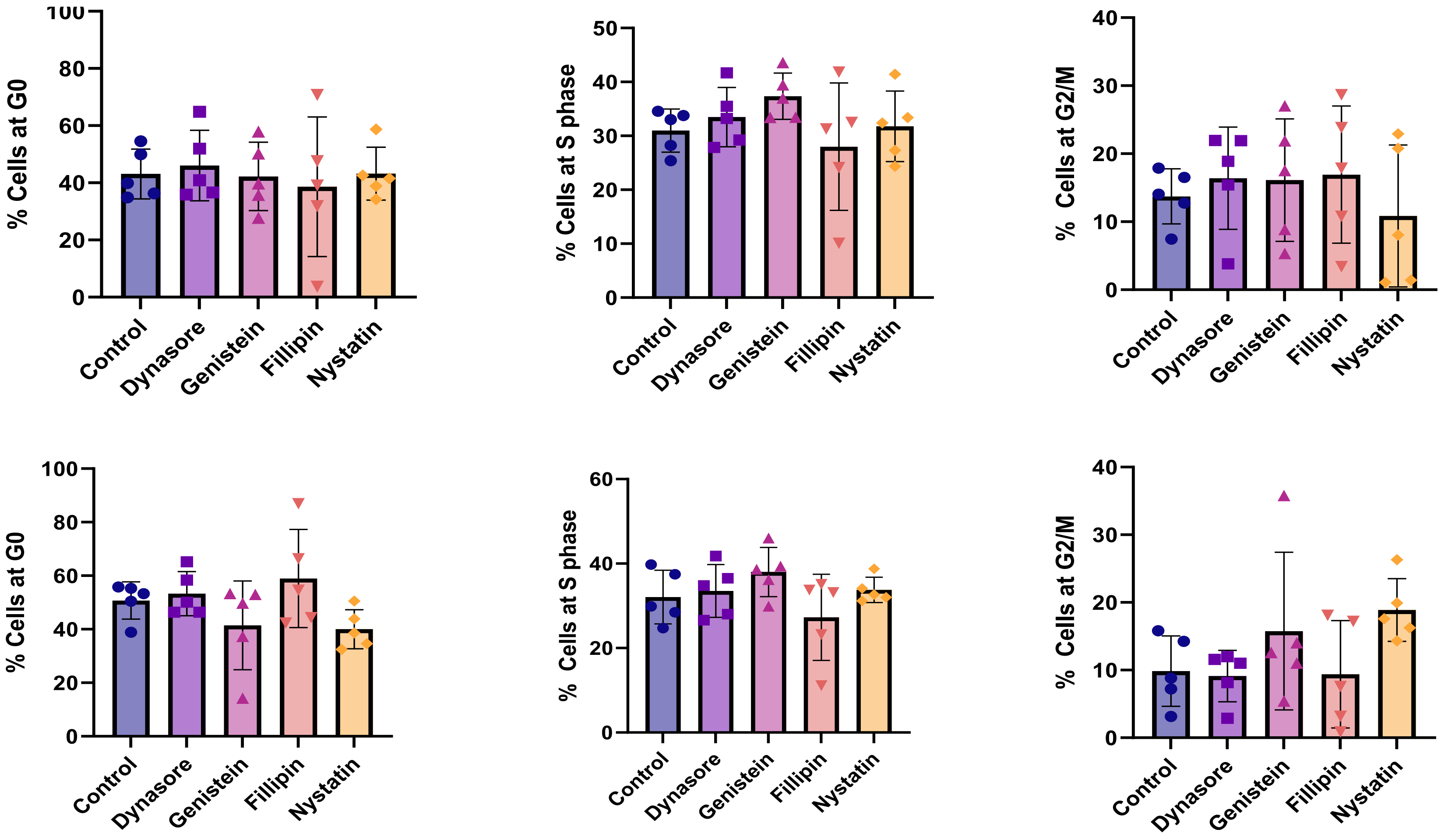

Supplemental Figure S2: Effect of Endocytosis inhibitors on cell cycle phase distribution. PC3ML were incubated with endocytosis inhibitors Dynasore, Genistein, Fillipin, or Nystatin during EV treatment. Treatment with endocytosis inhibitors did not change the relative proportion of cells at each phase of cell cycle.

Supplemental Figure S3

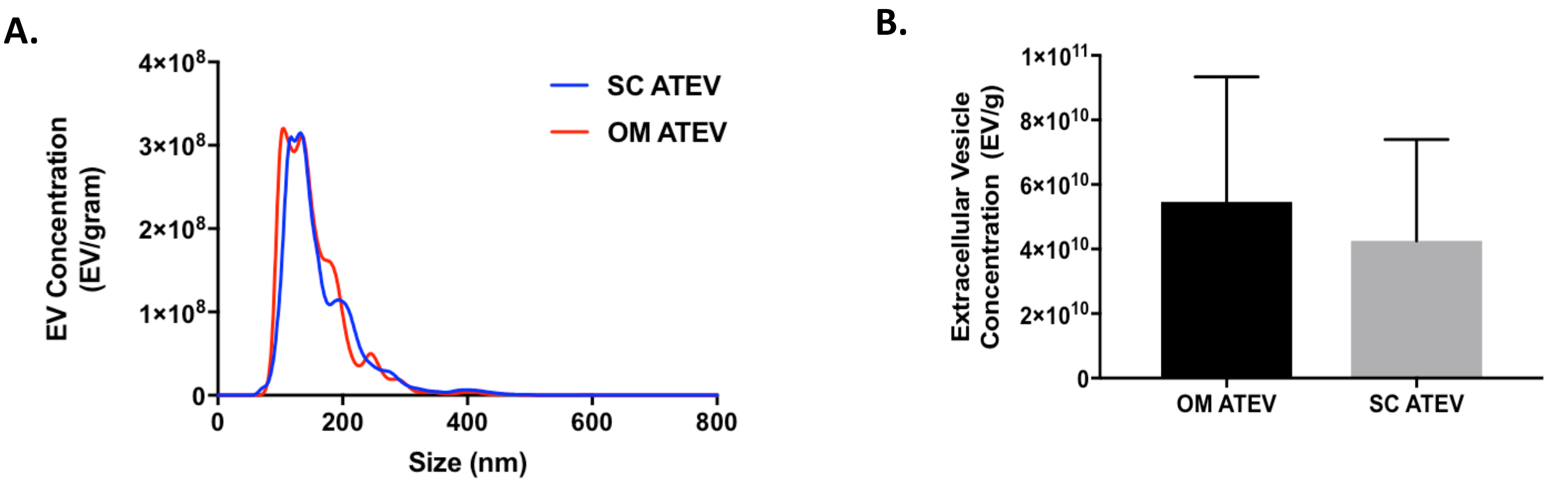

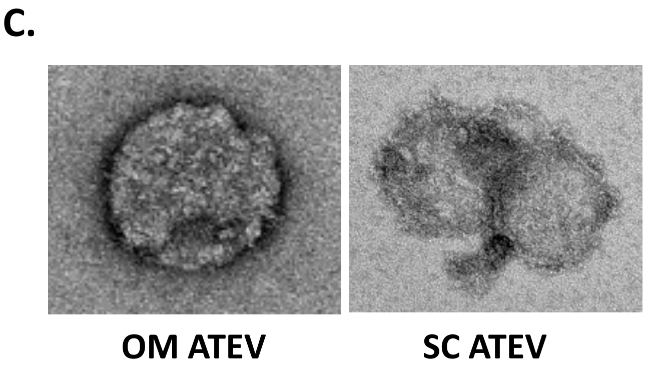

D. **Mascot Protein ID's – Common Exosome Markers**

Protein (n=6 samples In triplicate)	UniProt ID	Present
CD9	CD9_HUMAN	18/18
CD63	CD63_HUMAN	11/18
CD81	CD81_HUMAN	18/18
TSG101	TS101_HUMAN	0/18
Syntenin-1	SDCB1_HUMAN	18/18
Alix	PDC6I_HUMAN	18/18
CD82	CD82_HUMAN	3/18

Supplemental Figure S3: Characterization of EV isolated from omental and subcutaneous adipose tissue explants. **A:** Nanoparticle size distribution and concentration of extracellular vesicles (EV) isolated from omental (OM) and and subcutaneous (SC) adipose tissue. **B:** Nanosight analysis showed no significant difference in EV concentration for OM vs SC ATEV. Particle number was normalized to fat explant wet weight **C:** Representative micrographs of negative staining electron microscopy of ATEV from OM and SC adipose tissue **D:** EV markers were characterized via LC-MS/MS mass spectrometry for both OM and SC ATEV.
