# Supplementary material for: Adipose Tissue-Derived Extracellular Vesicles Contribute to Phenotypic Plasticity of Prostate Cancer Cells
Source: Int J Mol Sci. 2023 Jan 8;24(2):1229. doi: 10.3390/ijms24021229 (PMC9864182; doi:10.3390/ijms24021229)
Supplement: Supplementary file 1 [file ijms-24-01229-s001.zip › Supplemental Tables.pdf]

Supplemental Tables

miRNA	Target Genes							
**hsa-let-7i-5p**	AARSD1	CASP3	CSDE1	GYS1	MARS2	PLEC	SCYL1	THBS1
	ACP1	CCND1	CSNK1D	HMGA1	MED28	PLOD2	SEPTIN3	TLR4
	ADAMTS14	CDC25A	DAD1	HMGA2	MLLT1	POLD2	SIGMAR1	TPM2
	ADAMTS15	CDIPT	DHX57	HMOX1	MRM1	POLR2C	SLC1A4	TRIM71
	ADAMTS2	CDK6	DICER1	HYOU1	MRPS24	POM121	SLC25A1	TRMT1
	ADGRG1	CDKAL1	DOCK5	IFIT5	MRPS33	PPP1R7	SLC25A13	TTC9C
	AGO4	CEMIP2	DRD3	IFRD1	MTPN	PRDM1	SLC25A24	TUSC2
	AKAP8	CHMP2A	DSP	IGF2BP1	MTRR	PRIM1	SLC25A32	TYMS
	ANAPC1	CIAO2A	DUSP12	IGF2BP2	MYC	PRRC2A	SLC38A1	UGT8
	ATAD3B	COIL	DUSP23	IGF2BP3	NEDD4	PTGS2	SMC1A	UHRF1
	ATP6V0A1	COL1A1	EIF3J	IPO4	NF2	PXDN	SMOX	VCAN
	ATP6V1F	COL1A2	EIF4G2	ITGB3	NID1	RABGAP1L	SNAP23	VIM
	AURKB	COL4A1	F2	KCNJ16	NID2	RAS	SPARC	VPS39
	BCL2L1	COL4A2	FADS2	KLK10	NRAS	RBM19	SPCS3	WNT1
	BCL7A	COL4A5	FANCD2	KRAS	NXN	RDH10	SPRYD4	
	BMP2K	COL5A1	FNDC3A	KRT19	OTULINL	RHOB	SYPL1	
	BSG	COL5A2	GAK	LAMC1	P3H1	RHOG	TAF9B	
	CALCOCO2	COL8A1	GEMIN7	LIN28A	PDGFA	RPP38	TAGLN	
	CAPG	COMMD9	GRPEL2	LOXL2	PDGFB	RRP8	TGFBR1	
	CARHSP1	CRTAP	GTPBP3	LOXL4	PGRMC1	RTCA	TGFBR2	
**hsa-miR-126-3p**	CRKL	IRS1	PIK3R2	SLC45A3	SPRED1	TOM1	VCAM1	VEGFA
**hsa-miR-21-5p**	ACTA2	CDK6	GLCCI1	MTAP	PTEN	SLC16A10	TAGLN	
	APAF1	CDKN1A	HIPK3	NFIB	RECK	SOCS5	TCF21	
	BMPR2	CFL2	IL6R	PDCD4	RP2	SOD3	TGFBR2	
	BTG2	FAM3C	JAG1	PELI1	SERPINB5	SOX5	TIMP3	
	SGK3/SGK3	FAS	LRRFIP1	PIK3R1	SESN1	SPRY1	TNF	
	CDC25A	FASLG	MARCKS	PRRG4	SLC12A1	SPRY2	TPM1	
**hsa-miR-23a-3p**	ATAT1	GATD3A	IL6R	MET	POU4F2	SMAD3	TNFAIP3	
	CXCL12	HES1	LMNB1	NOTCH1	PTEN	SMAD4	TRIM63	
	FBXO32	HOXA11	MDH2	PLAU	SEPTIN3	SMAD5	TRPS1	
**hsa-miR-302d-3p**	ADAM9	CD83	FAM13B	KLHL12	MKRN1	PRKACB	TBC1D2	USP12
	ANKRD13B	CDK11B	FITM2	KPNA2	MYBL1	AS1	TEX30	VEGFA
	ANKRD52	CDK19	FYCO1	LATS2	NIN	RAB22A	TFAP4	VPS26A
	APP	CDKN1A	GBP3	LEFTY1	NIPSNAP2	RECK	TMEM14A	ZHX1
	ARHGEF3	CENPF	GLTP	LEFTY2	NR4A2	RELA	TMEM263	ZNF226
	BAZ1A	CFL2	GPSM2	LMNB1	NUP58	RNF149	TMEM9B	
	C9orf78	CMTM4	HERPUD1	LUC7L2	PACRGL	RPIA	TNFAIP1	
	CCND1	CNOT6	HSPA14	MARCHF4	PBK	SEPTIN2	TP63	
	CCND2	CYB5R4	INSIG2	MBNL2	PCGF5	SLC25A23	TRPS1	
	CD24	DKK1	KDM1B	MED28	PDIK1L	SLC35F6	TTC8	
	CD44	ERBB4	KIF23	MFSD4B	PHC2	STK4	TUSC2	
	CD46	ESR1	KLF13	MICA	PRC1	STX11	UBXN1	
**hsa-miR-378e**	CASP9	IGF1R	ODC1	PDPK1	SUFU	TUSC2		
**hsa-miR-411-5p**	SPRY4	TXNIP						
**hsa-miR-451a**	ABCB1	AKTIP	FBXO33	MIF				
**hsa-miR-548ar-5p**	ERBB2	MTA1						
**hsa-miR-612**	AKT2	TP53						

Supplemental Table S1: Target genes of HAMVEC EV miRNAs. The Ingenuity pathway analysis miRNA target filter tool was used to identify 318 experimentally validated target genes of the miRNA detected in both EV C and EV PIC

miRNA	Target Genes							
hsa-miR-155-5p	ABHD16A	CCN1	DNAJC19	JARID2	MYO10	PRAF2	SDCBP	TNFRSF10A
	AGTR1	CCND1	DPP7	LCLAT1	MYO1E	PRKCI	SH3BP4	TP53INP1
	AICDA	CD47	DSG2	LDOC1	NARS1	PTPRJ	SLA	TRAM1
	AMIGO2	CDK5RAP3	ETS1	LPL	NT5E	RAB23	SLC30A1	TRIM32
	ANKFY1	CEBPB	F2	LY6K	PDE3A	RAB27B	SMAD1	TRIP13
	ARFIP1	CHAF1A	FADD	MAF	PDLIM5	RAB34	SMAD2	TXNDC12
	ARFIP2	CLDN1	FADS1	MATR3	PHC2	RAB5C	SNAP29	TXNRD1
	ARID2	CSF1R	FAR1	MEIS1	PICALM	RAB6A	SOCS1	
	ARL10	CTLA4	FGF7	MET	PKN2	RAI14	SPI1	UFL1
	ARL5B	CTNNB1	FMNL2	METTL7A	PLXND1	RCN2	SYNE2	VAMP3
	ATG3	CUL4B	GNA13	MOSPD2	PMAIP1	RCOR1	SYPL1	WDFY1
	ATP6V1C1	CUX1	HSD17B12	MPZL1	PODXL	RHEB	TAB2	WEE1
	BACH1	CYP51A1	HSDL1	MSI2	POLE3	RHOA	TACSTD2	
	BET1	DCAF7	IKBKE	MTARC1	POLE4	RIPK1	TBCA	
	BRPF3	DHX40	IL13RA1	MYB	PPL	SATB1	TCF4	
	CBFB	DNAJB1	INPP5D	MYD88	PPP5C	SCAMP1	TM6SF1	

Supplemental Table S2: Target genes of miRNA found in HAMVEC EV PIC but not EV C. The Ingenuity pathway analysis miRNA target filter tool was used to identify experimentally validated target genes of the miRNA detected in only EV PIC. Only one miRNA detected in EV PIC alone (miR-155) was found to have experimentally validated target genes.

miRNA	Target Gene						
**miR-16-5p**	ABCF2	CDC14B	GALNT7	ITGA2	NPR3	RAB9B	TMEM109
	ABHD10	CDC25A	GFM1	JUN	NT5DC1	RAD51C	TMEM251
	ACP2	CDK5RAP	GFPT1	KCNN4	OGT	RAF1	TMEM43
	ACTR1A	CDK6	GNL3L	KIF23	OMA1	RARS1	TNFSF9
	ADSS2	CENPJ	GOLGA5	KITLG	OSGEPL1	RECK	TOMM34
	ALDH1A3	CEP63	GOLPH3L	KPNA3	PABIR3	RFT1	TPI1
	ANAPC16	CFL2	GPAM	LAMC1	PAFAH1B2	RHOT1	TPM3
	ANLN	CHEK1	GRB10	LAMTOR3	PANX1	RIDA	TPPP3
	ARHGDIA	CHORDC1	GRB2	LAMTOR5	PDCD4	RNASEL	TRMT13
	ARL2	CLDN12	GSTM4	LDAH	PDCD6IP	RNF144B	TXN2
	ASXL2	CREBL2	GTF2H1	LINC0066	PEDS1	RTN4	UBE2S
	ATF6	CRHBP	H3-3	LUZP1	PHKB	SEC24A	UBE4A
	ATG9A	CSHL1	HACE1	LYPLA2	PHLDB2	SERPINE2	UCP2
	BCL2	DIPK1A	HARS1	MAP2K1	PISD	SHOC2	UGDH
	BCL2L2	DMTF1	HDHD2	MAP2K4	PLAG1	SKAP2	UGP2
	BDNF	DNAJB4	HERC6	MAPK3	PLK1	SLC12A2	UTP15
	BMI1	DTD1	HMGA1	MCL1	PMS1	SLC16A3	VEGFA
	C17orf80	E2F3	HMOX1	MGAT4A	PNN	SLC25A22	VPS45
	C2orf74	ECHDC1	HPF1	mir-9	PNP	SLC35A1	VTI1B
	CA12	EGFR	HSDL2	MLLT1	PPIF	SLC35B3	WEE1
	CACNA2D1	EGLN2	HSP90B1	MLLT11	PPP2R5C	SLC38A1	WIPF1
	CADM1	EIF4E	HSPA1	MRPL20	PRIM1	SLC38A5	WNT3A
	CAPRIN1	F2	HYAL3	MSH2	PRIMPOL	SLC7A1	WT1
	CARD8	FGF2	IFNG	MYB	PSAT1	SPI1	YIF1B
	CCND1	FGF7	IFRD1	NAA15	PTGS2	SPTLC1	ZNF559
	CCND3	FGFR1	IGF1	NAPG	PURA	SQSTM1	ZNF622
	CCNE1	FLT3	IGF1R	NFIA	PWWP2A	SRPRA	ZYX
	CCNF	FNDC3B	IGF2R	NIPAL2	RAB21	SRPRB	
	CDC14A	GALNT13	IPO4	NOTCH2	RAB30	TIA1	
**let-7b-5p**	AARSD1	CDKAL1	Dst	IGF2BP3	NID2	RHOB	TGFBR1
	ACP1	CEMIP2	DUSP12	IPO4	NRAS	RHOG	TGFBR2
	ADAMTS14	CHMP2A	DUSP23	ITGB3	NXN	RPP38	THBS1
	ADAMTS15	CIAO2A	EIF3J	KCNJ16	OTULINL	RRP8	TLR4
	ADAMTS2	COIL	EIF4G2	KLK10	P3H1	RTCA	Tpm1
	ADGRG1	COL1A1	F2	KRAS	PDGFA	SCYL1	TPM2
	AGO4	COL1A2	FADS2	KRT19	PDGFB	SEPTIN3	TRIM71
	AKAP8	COL4A1	FANCD2	LAMC1	PGRMC1	SIGMAR1	TRMT1
	ANAPC1	COL4A2	FNDC3A	LIN28A	PLEC	SLC1A4	TTC9C
	ATAD3B	COL4A5	GAK	LOXL2	PLOD2	SLC25A1	TUSC2
	ATP6V0A1	COL5A1	GEMIN7	LOXL4	POLD2	SLC25A13	TYMS
	ATP6V1F	COL5A2	GRPEL2	MARS2	POLR2C	SLC25A24	UGT8
	AURKB	COL8A1	GTPBP3	MED28	POM121	SLC25A32	UHRF1
	BCL2L1	COMMD9	GYS1	MLLT1	PPP1R7	SLC38A1	VCAN
	BCL7A	CRTAP	HMGA1	MRM1	PRDM1	SMC1A	VIM
	BMP2K	CSDE1	Hmga2	MRPS24	PRIM1	SMOX	VPS39
	BSG	CSNK1D	HMGA2	MRPS33	PRRC2A	SNAP23	WNT1
	CALCOCO2	DAD1	HMOX1	MTPN	PTGS2	SPARC	
	CAPG	DHX57	HYOU1	MTRR	PXDN	SPCS3	
	CCND1	DICER1	IFIT5	MYC	RABGAP1L	SPRYD4	
	CDC25A	DOCK5	IFRD1	NEDD4	RAS	SYPL1	
	CDIPT	DRD3	IGF2BP1	NF2	RBM19	TAF9B	
	CDK6	DSP	IGF2BP2	NID1	RDH10	TAGLN	
**miR-30c-5p**	CARHSP1	COL4A1	IDH1	MYO10	PLEC	SLC38A2	TMED7

	CASP3	COL4A2	IFRD1	NAPG	PLOD2	SLC4A10	TMEM41B
	ADAMTS15	COL4A5	ITGA2	NCEH1	PNP	SLC4A7	TMEM59
	ADAMTS2	COL5A1	JUN	NCL	POGLUT3	SLC7A1	TMEM87A
	ADPGK	COL5A2	KRT7	NEUROD1	PPP3CA	SLC7A11	TNFAIP2
	ANPEP	COL8A1	KRT85	NID1	PPP3R1	SLC9A3R2	TNFRSF10B
	AP2A1	CPNE8	LAMC1	NID2	PRPF40A	SPARC	TNRC6A
	ATP2A2	CRTAP	LMNB2	NPR3	PTGFRN	STRN	TP53
	ATRX	DOCK7	LOXL2	NT5C3A	PTPA	STX1A	Tpm1
	BCL6	DST	LOXL4	NT5E	PTPRK	STX7	TRPS1
	BECN1	ELMOD2	LRRC8C	NUCB1	PTRH1	SYPL1	UAP1
	CBFB	F2	LTN1	NUFIP2	RAB27B	SYT4	UBE2I
	CCN2	FRG1	MAP4K4	P3H1	RAD23B	TGFBR1	VCAN
	CDCP1	FXR2	MAT2A	P4HA2	RBMS1	TGFBR2	WNT5A
	CEP72	GALNT1	MBNL1	PAFAH	RUNX2	THEM4	
	CHD1	GALNT7	MET	PDGFA	SEC23A	TMCO1	
	CNOT9	GNAI2	MLLT1	PDGFB	SEC62	TMED10	
	COL1A1	GPD2	MLLT11	PEX11B	SLC12A4	TMED2	
	COL1A2	HNRNPM	MPDU1	PGM1	SLC38A1	TMED3	
**miR-125b-5p**	ABTB1	BMF	CEBPG	GSS	KRT19	PPT2	TP53
	ACSS1	BMPR1B	CYP1A1	H3A/B	LIN28A	RABL6	TSPAN8
	ADAMTS1	CASP6	DDX19B	H4C1	LIPA	RBM8A	UBE2I
	AJUBA	CASP7	DICER1	HK2	MAN1A1	RHEBL1	UGT2B15
	ALOX5	CBFB	DIO3	ID1	MAP2K7	SCD	UGT2B17
	ANAPC16	CBLN2	DUS1L	ID2	MAZ	SGPL1	UGT2B28
	APLN	CBX7	E2F3	ID3	MYD88	SH3BP4	UVRAG
	ARID3A	CCR5	ELAVL1	IGFBP3	OSBPL9	SMO	VSIR
	ARID3B	CDC25A	ENTPD4	IKZF4	PCDHB10	ST18	ZNF385A
	ATP6AP1L	CDH5	ERBB2	IL1RN	PCTP	TAFA1	
	B3GALT4	CDK6	ERBB3	JARID2	PERP	TENM2	
	BAK1	CDKN2A	GPR160	KLF13	PIGR	TOR2A	
**miR-29b-3p**	ACVR2A	COL1A1	DNMT3A	HMGN3	NAV3	PURA	TGFBR2
	ADAMTS14	COL1A2	DNMT3B	INSIG1	NID1	RERE	TNFAIP3
	ADAMTS15	COL3A1	DST	KCTD3	NID2	SHROOM2	Tpm1
	ADAMTS2	COL4A1	DUSP2	KLF4	P3H1	SP1	TRIM9
	ARPC3	COL4A2	EOMES	LAMC1	PDGFA	SPARC	TUBB2A
	BACE1	COL4A5	FAM3C	LOXL2	PDGFB	SRSF10	VCAN
	CAV2	COL5A1	FBN1	LOXL4	PIK3R1	TBX21	YY1
	CD276	COL5A2	FRAT2	MAPRE2	PLEC	TCL1A	ZFP36L1
	CDC42	COL5A3	GAS7	MCL1	PLOD2	TDG	
	CDK6	COL8A1	GMFB	MLF1	PPIC	TET1	
	CNOT8	CRTAP	GPR37	MYBL2	PPM1D	TGFB3	
	COL15A1	DCP2	HDAC4	NASP	PTEN	TGFBR1	
**miR-122-5p**	AACS	ATP1A2	CS	FUNDC2	MEP1A	RAB11FIP1	TMEM50B
	ADAM17	BACH2	DSTYK	G6PC3	NCAM1	RAB6B	TPD52L2
	AKT3	BCKDK	DUSP2	GALNT10	NDRG3	RABIF	TRIB1
	ALDOA	BCL2L2	EGLN3	GPX7	NFATC1	SLC35A4	TRPV6
	ANK2	CCNG1	ENTPD4	GYS1	NFATC2IP	SLC7A1	TTYH3
	ANXA11	CD320	FAM117B	HJV	NUMBL	SLC7A11	UBAP2
	AP3M2	CERS6	FOXJ3	MAPK11	OSMR	TBX19	XPO6
	ATP11A	CLDN18	FOXP1	MECP2	PALM	TMED3	
**miR-145-5p**	ACBD3	CLINT1	FLI1	LINC0033	MYO5A	RTKN	USP46
	AHNAK	DDR1	FSCN1	MAPK7	NDUFA4	SOX9	VASN
	C11orf58	DFFA	IGF1R	MDFI	PADI1	SPTB	
	CBFB	DTD1	IRS1	MDM2	PARP8	SWAP70	
	CCDC25	EIF4E	KLF4	MITF	PPP3CA	TPM3	
	CCNA2	EIF4EBP2	KLF5	MMP1	RAB27A	TYR	
	CDK4	F11R	KRT7	MUC1	RASA1	TYRP1	
	CES1	FBXO28	LAMP2	MYC	ROBO4	UNG	
**miR-93-5p**	APP	BNIP2	E2F1	ITCH	NCOA3	RBL2	TNF

	ARID4B	CAMTA1	E2F2	JAK1	PAK5	RUNX1	TP63
	BAMBI	CCND1	E2F3	MAP3K12	PKD2	S1PR1	TUSC2
	BCL2	CDKN1A	EGR2	MEF2D	PPARG	STAT3	VEGFA
	BCL2L11	CREB1	ESR1	MICA	PTEN	TBC1D2	VIM
	BLACAT1	CRIM1	HBP1	MMP3	PURA	TGFBR2	ZBTB7A
	BMPR2	CXCL8	HIPK3	MYLIP	RB1	TLR7	
**miR-21-5p**	ACTA2	CDK6	GLCCI1	MTAP	PTEN	SLC16A10	TAGLN
	APAF1	CDKN1A	HIPK3	NFIB	RECK	SOCS5	TCF21
	BMPR2	CFL2	IL6R	PDCD4	RP2	SOD3	TGFBR2
	BTG2	FAM3C	JAG1	PELI1	SERPINB5	SOX5	TIMP3
	SGK3	FAS	LRRFIP1	PIK3R1	SESN1	SPRY1	TNF
	CDC25A	FASLG	MARCKS	PRRG4	SLC12A1	SPRY2	TPM1
**miR-23a-3p**	ATAT1	GATD3A/B	IL6R	MET	POU4F2	SMAD3	TNFAIP3
	CXCL12	HES1	LMNB1	NOTCH1	PTEN	SMAD4	TRIM63
	FBXO32	HOXA11	MDH2	PLAU	SEPTIN3	SMAD5	TRPS1
**miR-25-3p**	BCL2L11	CDKN1A	FBXW7	ITGA5	MAPRE1	OSBPL8	RFFL
	BMPR2	CDKN1C	HIPK3	ITGB3	MYLIP	PCGF1	VSNL1
	CCNE2	ENPP6	IKZF1	MAP2K4	OSBPL2	PTEN	ZEB2
**miR-181a-5p**	AICDA	CDKN1B	GATA6	KRAS	NOTCH4	TIMP3	
	BCL2	CDX2	GRIA2	MMP14	PLAG1	TRA	
	CD69	ESR1	HOXA11	NLK	TCL1A	VSNL1	
**miR-26b-5p**	BAK1	EPHA2	HPGD	MAP2	PLAG1	PTGS2	SMAD1
	CHORDC1	EZH2	HTR1A	PHF6	PTEN	RB1	TGFBR2
**miR-129-5p**	AGO3	CAMTA1	ETV6	GALNT1	PDS5A	TNPO1	ZFP91
	BMPR2	DNMT3A	FNDC3B	HIF1A-	SOX4	TP53INP1	
**miR-223-3p**	CNTN4	LMO2	NFIA	SCN3A	STMN1		
	IRS1	MEF2C	RHOB	STAT3	VIM		
**miR-150-5p**	AICDA	BACH1	CSF1R	MALAT1	PDGFB		
	Akt	CEBPB	EGR2	MYB	VEGFA		
**miR-199b-5p**	ALOX5AP	ETS1	LAMC2	PDCD4	SIRT1		
	DYRK1A	HIF1A	MYH9	SET	SNHG29		
**miR-130a-3p**	ATG2B	DICER1	MAFB	SMAD4	ZFPM2		
	CSF1	HOXA5	MEOX2	TAC1			
**miR-10a-5p**	APAF1	HOXD10	LINC00324	PTEN			
	HOXA1	KLF4	NF1	USF2			
**miR-126-3p**	CRKL	PIK3R2	SPRED1	VCAM1			
	IRS1	SLC45A3	TOM1	VEGFA			
**miR-99a-5p**	EPDR1	FGFR3	MTOR	RPTOR			
	FGF16	IGF1R	PLK1	SMARCA5			
**miR-214-3p**	ATF4	GPD1	LINC00324	PTEN			
	FGF16	ING4	POU4F2	SCN3A			
**miR-148a-3p**	CCKBR	DNMT3B	HOTAIR	RPS6KA5			
	DNMT1	DUSP1	NR1I2	Tnxa-ps1			
**miR-142-3p**	ADCY9	BCL2L1	BCLAF1	C9orf72	LIFR	PRKCA	TARDBP
**miR-494-3p**	FGF16	HMOX1	KIT	PTEN	SCN3A	VSNL1	
**miR-22-3p**	BMP7	ESR1	MAX	PPARA	SRF		
**miR-191-5p**	Ccl9	CRP	IL6	TLR3			
**miR-451a**	ABCB1	AKTIP	FBXO33	MIF			
**miR-612**	AKT2	TP53					
**miR-411-5p**	SPRY4	TXNIP					
**miR-144-3p**	ENPP6						
**miR-145-5p**	ABRACL						
**miR-574-3p**	TP63						
**miR-342-3p**	MTDH						

Supplemental Table S3: Target genes of miRNA detected in SC and OM ATEV. The Ingenuity pathway analysis miRNA target filter tool was used to identify experimentally validated target genes of the miRNA detected in miRNA detected in SC and OM ATEV.
